# Animal influence on water, sanitation and hygiene measures for zoonosis control at the household level: A systematic literature review

**DOI:** 10.1371/journal.pntd.0006619

**Published:** 2018-07-12

**Authors:** Francisco Matilla, Yael Velleman, Wendy Harrison, Mandy Nevel

**Affiliations:** 1 Department of Pathobiology and Population Sciences, The Royal Veterinary College, London, United Kingdom; 2 Policy and Campaigns Department, WaterAid, London, United Kingdom; 3 Faculty of Medicine, School of Public Health, Imperial College, London, United Kingdom; University of Minnesota, UNITED STATES

## Abstract

**Introduction:**

Neglected zoonotic diseases (NZDs) have a significant impact on the livelihoods of the world’s poorest populations, which often lack access to basic services. Water, sanitation and hygiene (WASH) programmes are included among the key strategies for achieving the World Health Organization’s 2020 Roadmap for Implementation for control of Neglected Tropical Diseases (NTDs). There exists a lack of knowledge regarding the effect of animals on the effectiveness of WASH measures.

**Objectives:**

This review looked to identify how animal presence in the household influences the effectiveness of water, hygiene and sanitation measures for zoonotic disease control in low and middle income countries; to identify gaps of knowledge regarding this topic based on the amount and type of studies looking at this particular interaction.

**Methods:**

Studies from three databases (Medline, Web of Science and Global Health) were screened through various stages. Selected articles were required to show burden of one or more zoonotic diseases, an animal component and a WASH component. Selected articles were analysed. A narrative synthesis was chosen for the review.

**Results:**

Only two studies out of 7588 met the inclusion criteria. The studies exemplified how direct or indirect contact between animals and humans within the household can influence the effectiveness of WASH interventions. The analysis also shows the challenges faced by the scientific community to isolate and depict this particular interaction.

**Conclusion:**

The dearth of studies examining animal-WASH interactions is explained by the difficulties associated with studying environmental interventions and the lack of collaboration between the WASH and Veterinary Public Health research communities. Further tailored research under a holistic One Health approach will be required in order to meet the goals set in the NTDs Roadmap and the 2030 Agenda for Sustainable Development.

## Author summary

Neglected Tropical Diseases (NTDs) affect the health and economies of populations globally. Many of these diseases are zoonotic, occurring as a consequence of the interaction between humans and animals, particularly at the household level in low- and middle-income countries. Based on the WHO Global Strategy to accelerate and sustain progress on NTDs, including zoonoses, through improvement in sanitation, hygiene and water, this review identifies existing published studies examining the interaction between water, sanitation and hygiene elements, animals and zoonosis transmission within the household. Only two out of 7588 studies screened met the criteria. They showed the relevance of animal influence in the effectiveness of WASH measures, as well as the difficulties of designing studies that look at this particular interaction. A synthesis of several studies analysed in the second selection stage of the review shows a significant relationship between animal and WASH factors for disease transmission. It also shows certain contradictions regarding the importance of key risk factors for some diseases across studies. It is therefore crucial to carry out further studies showing the interaction between animals and water, hygiene and sanitation measures within the household to improve these control measures and reduce zoonotic neglected tropical disease transmission.

## Introduction

### Neglected tropical diseases and zoonoses

Neglected tropical diseases (NTDs) are a group of communicable diseases estimated to affect over a billion people globally, particularly those with least economic resources, access to health care, good nutrition, clean water and sanitation facilities; the weak political influence of affected groups as well as the complex nature of these diseases has resulted historically in a lack of attention and resources, precipitating the use of the term “neglected”[1]. This has been acknowledged by the World Health Organisation (WHO) and a global Roadmap was released in 2012 to focus on reducing the burden of 17 NTDs. This “Roadmap for Implementation” [2] includes five ‘key strategies to combat NTDs by 2020’ of which one aims to improve veterinary public health at the human–animal interface, and another emphasises the provision of safe and clean sources of water and effective sanitation infrastructure, and ensuring appropriate hygiene practices (WASH) [3]. The Roadmap, together with the 2015 WHO global strategy on WASH and NTDs [4], espouses a holistic approach to disease control and elimination.

The new global development framework enshrined in the Global Goals of the United Nations’ 2030 Agenda for Sustainable Development [5] sets out a One-Health approach to poverty, inequalities, health and the environment, in contrast with the siloed structure of the previous Millennium Development Goals (MDGs), whose agenda ended in 2015. Global Goal 3 within this agenda sets ambitious targets for improving health and wellbeing, including NTDs, and acknowledges the importance of addressing social and environmental determinants of health [6]. A One Health approach that addresses the animal-human interface and defines disease control strategies that enhance livelihoods and reduce poverty can contribute to the achievement of the Global Goals, but also represents a departure from current prevailing practices. Further knowledge on effective programming approaches is therefore urgently needed.

Several of the NTDs are zoonotic diseases—infections transmitted between animals and humans, and are therefore referred to as Neglected Zoonotic Diseases (NZDs). These include cysticercosis, rabies, echinococcosis, foodborne trematodiases, zoonotic African trypanosomiasis and schistosomiasis. Several of these are related to WASH elements in terms of prevention and/or treatment. Other diseases recognised by WHO in its “Research Priorities for Zoonoses and Marginalized Infections” include toxoplasmosis, cryptosporidiosis and bacterial zoonoses, for which improved sanitation has proven effective in reducing transmission [3]. The global burden of these zoonotic diseases is considerable. Cystic echinococcosis causes, on average, the loss of 2 million annual disability-adjusted life years (DALYs), with associated costs rising up to US$ 3 billion for human treatment and livestock industry losses [7]. *Taenia solium*, the causal agent of taeniasis and cysticercosis, is responsible for an estimated cost of 2.8 million DALYs globally [8]. Mortality due to cysticercosis in humans increased by 58% between 1990 and 2010 [9], and the disease is estimated to affect over 50 million people globally, causing up to 30% of all epilepsy cases [10]. Zoonoses are estimated to contribute to up to 10% of the total DALYs lost, and 26% of DALYs lost due to infectious diseases in low income countries [11]. Zoonoses affect human health directly, but by affecting animal health, they can also cause important economic losses and limitations for affected rural communities that depend on animals for working fields, transportation, as a source of protein and as a source of income when sold in local markets [12]. For example, cysticercosis has been reported to cause $12,6 million in annual losses in Cameroon [13], $150 million in India [14] and 18.6 to 34.2 million US dollars in East Cape, South Africa [15].

### One Health approach to NZDs

These zoonotic diseases are neglected due to the relatively low mortality associated with them, their tendency to affect predominantly poor and marginalised populations, and the complex, intersectoral measures required to control them, which include community infrastructure and capacity building, health promotion programmes, improved diagnostics and treatment, vaccination and prevention programmes and policy adaptation at local, regional, national and international level [11]. Zoonotic pathogens have complex life cycles that commonly include different phases in human hosts, animal hosts and the environment before completion. Overlooking one or more of these three elements facilitates the perpetuation of the cycle, and with it, reinfection. A One Health approach to controlling zoonotic transmission is needed, considering animals, people and the environment in a comprehensive approach to public health. Since zoonoses are influenced directly and indirectly by multiple factors, focusing solely on transmission routes wrongfully overlooks socio-cultural, economic, anthropological and ecological elements that may affect transmission as well as delivery of control programmes.

The need for intersectoral control measures is especially evident in low income countries [16], where the rural population accounts for an average of 69% of the total [17]. Not only do poor, rural communities have fewer resources and less access to healthcare, they also possess less political influence and power than other population groups to demand services and resources from government authorities [18–20]. A One Health approach helps create resilient solutions for disease transmission by setting measures that can be implemented in the long term by community and government action, meeting the objectives for sustainability set by the Sustainable Development Goals [21]. In poor, rural settings, smallholder animal production of indigenous species of pigs, poultry and ruminants is dominant [22], and hence human and animal interaction within the household is more common in these settings, requiring special attention to this interaction in the control of zoonotic diseases [23]. However, given the dependence of rural households on animals as a major source of livelihood and as an alternate source of income in emergencies, certain measures that may support disease control objectives may not be feasible in practice [24]. For example, pig-corralling is recommended as a main method for control of cysticercosis, and hence programmes may be put in place to improve this practice amongst farmers [25]. However, for many households and communities in middle-low income countries, this is not economically feasible [26], since this would require the family to assume the added cost of feeding the pigs, instead of allowing the animals to forage for themselves [27]. Similarly, protecting water sources from animal access prevents contamination of water for human use with animal faeces and secretions. However, the need to provide livestock and humans with sufficient clean water from a protected source poses a challenge for many communities [28].

A One Health approach can help identify such multi-factorial elements and avoid omitting valuable programme components, including human, environmental and animal factors. Human behaviour factors such as conflict, migration and socio-cultural practices, shape disease patterns, due to relocation, high human density and reduced hygiene levels [29]. Similarly, economic and agricultural development will reshape the land and demands of society, changing animal farming and animal product consumption practices, increasing the risk of food-borne disease transmission and zoonotic influenza [30]. An example of an animal factor to consider is how wildlife reservoirs can help perpetuate infective cycles within local livestock. This poses a great challenge for zoonotic disease control in pastoral communities due to the difficulty of limiting direct and indirect interaction between wildlife and livestock species [30, 31]. Additionally, ecological factors like climate change and deforestation have a direct impact on the distribution of vector-borne diseases by altering the habitats of the vector and reservoir species, as well as allowing vectors to sustain their life cycle in new areas due to a rise in average temperatures, leading to emergence and re-emergence of these diseases in new parts of the world [30, 32]. Another example of One Health approaches helping to tackle ecological problems can be found in the reuse of animal excreta as crop manure, as incorrect use can lead directly to disease transmission through contact and clothes and indirectly through water contamination [33]. Use of animal excreta as crop manure can also alter the chemical properties of the soil, endangering the environmental sustainability of the area, and subsequently increasing the exposure of humans and animals to contaminated sources of infection [33]. Authors like Nguyen-Viet, Zinsstag and Charron propose an integration method as a solution for optimising the use of human and animal excreta as manure, by combining cross-sectoral knowledge and stakeholder engagement under a One Health framework [33, 34]. Such a framework enables the implementation of sustainable control strategies for NZDs in countries where economic resources are scarce.

### One Health challenges for WASH programmes

Water, sanitation and hygiene (WASH) programmes can plausibly contribute to control of zoonotic disease given the knowledge about pathogen transmission cycles, through provision of sanitation infrastructure that safely removes human and animal faecal waste from the human environment, provision of clean water sources, and improvement of hygiene practices at the community and household level [4]. The WHO WASH and NTDs strategy is a step towards developing collaboration between WASH and NTDs programmes, both of which reference integration of control measures, but do not offer specific guidance or methods of monitoring on collaboration between the sectors [4]. However, the much needed guidance to encourage a One Health approach through engagement of other sectors such as agriculture and veterinary public health is not included in the remit of the WASH and NTDs strategy [5, 35]. The positive relationship between WASH programmes and reduction of NTDs incidence has been proven, yet many of these programmes still lack the multifactorial approach needed to cover the impact of other elements that affect disease transmission [36], such as animal presence within the household and human-animal interaction. Because of this, there are limitations to understanding why WASH programmes may not result in the expected disease control outcomes and how they can be optimized. No systematic research has been done to date on the impact of demand-side sanitation programmes on NZDs transmission [3].

Although the evidence base on the interaction of animals with sub-standard sanitation facilities is weak, it is plausible that the presence of free-roaming household animals alongside conditions of open defecation or poor containment of faeces can contribute to intensified disease transmission [37]. As mentioned in the WHO WASH and NTDs Strategy [4], and as several authors argue [36, 38–40], it is necessary to gather more information regarding WASH-related interventions and disease burden reduction. This is particularly relevant for zoonotic diseases, as, out of the existing reviews relating to WASH and disease burden, few focus specifically on zoonotic diseases. Those that do, often disregard the presence of animals in the household and its impact on the effect of WASH interventions on zoonotic disease. There is need to identify these linkages and knowledge gaps that require further study. The aim of this work was to conduct a systematic review to identify the existing published data, on how the presence of animals in the household impacts the efficacy of WASH interventions for zoonotic disease control.

The objectives of this review were: to identify how animal presence in the household influences the effectiveness of water, hygiene and sanitation measures for zoonotic disease control in low and middle income countries; to identify gaps of knowledge regarding this topic based on the amount and type of studies looking at this particular interaction.

## Material and methods

### Protocol

A review protocol was designed to inform and direct the review steps before conducting the systematic review. The protocol was designed based on the guidelines given by “CRD’s guidance for undertaking reviews in health care” and the “WHO Handbook for Guideline Development” [41, 42], as well as example systematic review protocols found in various academic sources, approved by peer academic experts. The complete protocol can be found in Text S1.

### Search strategy

Three databases were used: Medline, Web of Science and Global Health. These were chosen based on other systematic reviews conducted in the area of sanitation, hygiene and NTDs [43–45], and on expert academic advice solicited by the authors. The three databases were systematically searched for publications dating 1980 to 30^th^ April 2016.

The search terms relative to WASH were chosen based on other WASH literature reviews and scientific articles. Animal terms were selected based on literature and expert advice, including those species most likely to interact with humans within the household, in low- and middle-income countries. The terms were then divided into four pools:

1. Water, hygiene and sanitation: *{[latrine]*, *[toilet]*, *[water]*, *[water supply]*, *[water treatment]*, *[education]*, *[borehole]*, *[standpipe]*, *[rainwater]*, *[sanitary engineering]*, *[pit]*, *[open defecation]*, *[open urination]*, *[shower laundry]*, *[hygiene]*, *[detergent]*, *[soap]*, *[risk factor]*, [excre*], [faec*], *[fecal]*, *[feces]*, *[hand washing]*, *[handwashing]*, *[waste management]*, *[waste disposal]}*
2. Animals: *{[horse]*, *[pig]*, *[chicken]*, *[turkey]*, *[cow]*, *[dog]*, *[cat]*, *[bovine]*, *[ovine]*, *[porcine]*, *[poultry]*, *[corralling]*, *[farming]*, *[buffalo]}*
3. Disease: *{[ntds]*, *[nzd]*, *[neglected zoonotic disease]*, *[ntd]*, *[neglected tropical disease]*, *[taenia solium]*, *[cysticercosis]*, *[taeniasis]*, *[pig tapeworm]*, [trypanosom*], *[hat]*, *[nagana]*, [echinococc*], *[hydatidosis]*, [schistosom*], *[snail fever]*, [foodborne trematod*], *[fbt]*, [chlonorch*], *[distomatosis]*, *[liver rot]*, [opisthorch*], [paragonim*], *[lung fluke]*, [toxoplasm*], [cryptosporid*], [crypto*], [brucell*], *[anthrax]*, *[anthracis]*, [leptospir*], [shigell*], *[Escherichia coli]*, *[mycobacterium bovis]*, *[m*. *bovis]}*
4. Location: The location terms consisted of the names of all the countries included in the High-Middle, Low-Middle and Low Income countries as defined by the World Bank [46–48].

The terms amongst pools were combined by the Boolean operator “OR”, while those between pools were combined by the Boolean operator “AND”.

Diseases chosen for the terms were based on the list of neglected zoonotic diseases described in the WHO NTDs Roadmap [2]. The results obtained were sorted by “author” in descending order. Studies were selected through a three-stage process, first by title and abstract screening, then by full text analysis, based on the selection criteria for each stage, and finally by a quality control checklist. References were managed with the use of reference management software EndNote X7.

### Inclusion/Exclusion criteria

For the first stage, title and abstract screening, studies were included if the abstract mentioned a zoonotic disease term together with a WASH term, if a full text version was available and if the article was published in English or Spanish. Studies not meeting these requirements, and review articles, were excluded.

The full text versions of studies selected in this first stage were retrieved and analysed for further selection. In this second stage, articles that did not quantify burden of disease in human or animal populations, did not analyse the role of animals in zoonosis transmission in relation to WASH measures, or did not meet the requirements of the quality check described in the protocol, were excluded from the review. The type of study and its design were not deemed to be crucial inclusion/exclusion criteria, due to a low number expectancy of final study retrieval.

### Quality assessment

Studies selected for the last stage of the systematic review were analysed using a quality checklist based on the guidelines for public health studies from the National Institute for Health and Clinical Excellence [49].

### Data extraction and synthesis

Articles included in the full text review were subjected to data extraction based on the protocol, with special attention to the study population regarding burden of disease, the diagnostic method used, the WASH measures in place, description of animal presence within the household, and the statistical analysis approach taken by the study. Due to the consideration of various types of studies in the inclusion criteria and the expected low count of final studies making the last selection, pooling was not deemed possible. Therefore, a narrative approach was chosen for addressing data synthesis. Zoonotic diseases in which WASH measures play a relevant role in control were included in the analysis and synthesis of the results, as long as the selected study included it in its own analysis, even if said diseases were not considered to be neglected by inclusion in the WHO reference list.

## Results

### First screening

**Fig 1:**
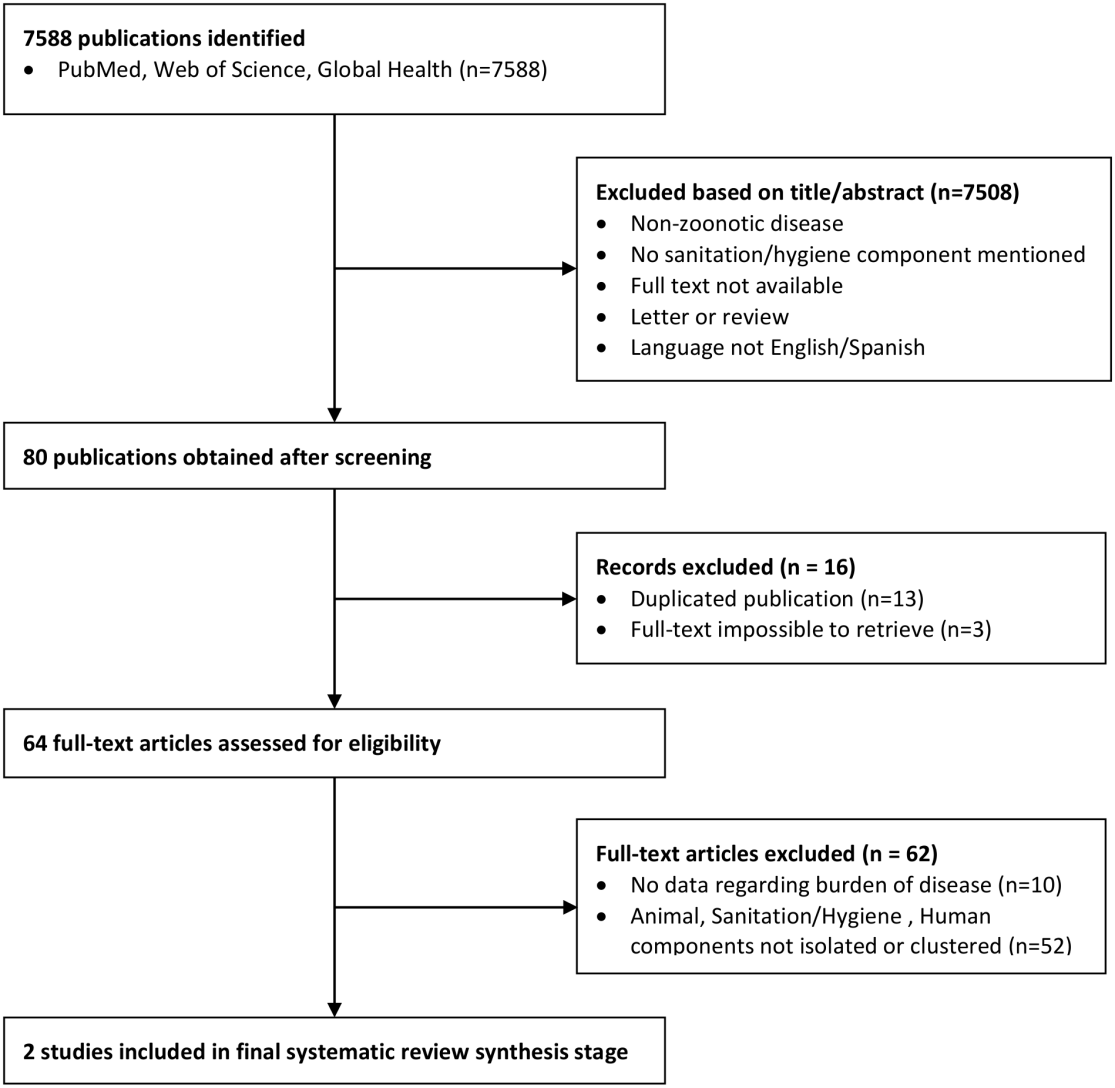
Flow chart describing the systematic selection process.

Seven thousand five hundred and eighty-eight (n = 7588) studies where obtained after introducing the search terms into the three databases (Fig 1). Screening of titles and abstracts retrieved a total of 80 studies (n = 80) meeting the inclusion criteria for the first stage of the review: 46 from Medline, 28 from Global Health, and six from Web of Science. Of these 80, 13 were duplicates and three were unable to be retrieve in full-text form and were therefore discarded. The total number of articles selected for the next stage of the review was 64.

### Second screening

Full text for the remaining 64 articles was obtained, analysed and considered for review inclusion. After data extraction and analysis, two articles [50, 51] were identified that quantified the burden of disease in humans or animals and analysed the role of animals in zoonosis transmission in relation to WASH measures, hence meeting the final inclusion criteria as set out in the protocol. Due to the low count of studies included in the final review, the 64 articles analysed in this phase were summarised in the form of tables that show the research tendencies when addressing WASH and NZDs. The complete list with the main data extracted from each one can be found in Table 1, including location, type of study, number of participants in the study, disease of interest, diagnostic test used to address presence of disease, WASH and animal component studied, the type of statistical method used for the analysis, and a summary of the results of the study.

**Table 1:** Identified studies results table summary.

First author	Year	Location	Study design	Participants/ Samples	Disease	Diagnostic	WASH component	Animal component	Data analysis	Results
**Abu EK et al.** [52]	2015	Central Region, Ghana	Cross-sectional	390 humans between 10–100 years	Toxoplasmosis	ELISA	Hand washing Water source	Cat litter Cats in household Dogs in household	X^2^ MR	Seroprevalence was 85%, risk factors identified included cat presence and unsafe drinking water source.
**Agampodi et al.** [53]	2015	Central Sri Lanka	Case-control	111 human cases, 222 controls	Leptospirosis	MAT-PCR	Water source	Animal farms Animals IH Dog/Cattle handling	UR MR	Risk factors identified included dog presence and cattle presence at home. Piped water acted as a protective factor.
**Ahmad et al.** [54]	2015	Punjab, Pakistan	Cross-sectional	413 sheep, 419 goats	Toxoplasmosis	ELISA	Hygienic condition Water source	Presence of cats	UR	Prevalence was 18.16% in sheep and 14.32% in goats. Risk factors included poor hygienic conditions, presence of cats, extensive farming practice and usage of outdoor water source.
**Ahmad et al.** [55]	2014	Northern Punjab, Pakistan	Cross-sectional	400 cattle, 422 buffalo	Toxoplasmosis	ELISA	Hygienic condition Water source	Cats in the vicinity Farming system	UR MR	Prevalence was 19.75% in cattle and 15.16% in buffaloes. Risk factors included poor hygiene, extensive farming and presence of cats.
**Aluja et al.** [56]	2013	Guerrero-Oaxaca-Chiapas, Mexico	Cohort study	1204 pigs	Cysticercosis	ELISA Tongue Ultrasound	Educational program	NA	Percentage	Prevalence reduced from 13.7% to 0% with a 3-year sustained effort.
**Alvarado-Esquivel et al.** [57]	2008	Durango, Mexico	Cross-sectional	463 adults	Toxoplasmosis	Serology	Drainage at home Housing condition	Cleaning cat feces	BR MR	Prevalence varied from 14.8% to 35.8%. Relevant risk factors included good home drainage, cat faeces disposal practices and consumption of turkey and squirrel.
**Andrade et al.** [58]	2013	Rio Grande do Norte, Brazil	Cross-sectional	930 sheep	Toxoplasmosis	ELISA	Water source	Presence of cats	UR MR	Prevalence was 22.1% overall. Risk factors included cat presence together with running water as a water source.
**Assana et al.** [59]	2010	Mayo-Danay, Cameroon	Cross-sectional	398 pigs	Cysticercosis	ELISA	Latrine availability Latrine use	Free-roaming pigs Pig pen availability	UR	Prevalence was 26.6%. Open defecation was practiced in 76% of the households. Risk factors identified included free roaming of pigs.
**Awadallah et al.** [60]	2015	Egypt	Cross-sectional	130 dog fecal samples; 150 human fecal samples; 150 human serum samples	Toxocariasis	ELISA Macroscopy	Hand washing	Raising dogs	UR	Prevalence was 30% in dogs and 24% in humans. Risk factors included raising dogs and not washing hands before meals.
**Bardosh et al.** [61]	2014	Phongsali, Lao PDR	Ethnographic	57 villagers	Zoonotic helminthiases	NA	Hygiene practices Latrine presence Latrine use	Farming system Pig management	Manual coding	Risk behaviours were mediated by limited market access, consumption of raw pork and poor latrine coverage.
**Boukary et al.** [62]	2010	Niamei, Niger	Retrospective and longitudinal survey	819 cattle, 7 sheep, 1 goat, 20 camels	Bovine tuberculosis	Bacteriology Macroscopy	Disinfectant use Hygienic condition	Presence of sick animals	PR UR	Prevalence was 0.19% in cattle, 0.11% in camels, 0.001% in sheep and 0.0006% in goats. Relevant risk factors identified included consumption of unpasteurized milk and lack of hygiene within households.
**Braae et al.** [63]	2015	Mbeya-Mbozi, Tanzania	Case-control	107 household pigs	Cysticercosis	Questionnaire/ observational survey	Type of latrine	Farming system Free-roaming piglets	UR	Porcine cysticercosis was associated with absence or open latrine as opposed to an enclosed latrine.
**Bulaya et al.** [50]	2015	Katete, Zambia	Comparative cross-sectional	64 pre-intervention pigs; 89 post-intervention pigs	Cysticercosis	ELISA	Presence/usage of latrines	Farming system Pig herd size	UR Wald test	Results explained further in the next manuscript section.
**Chaabane-Banaoues et al.** [64]	2015	Tunisia	Cross-sectional	1095 dog fecal samples	Echinococcosis	PCR	NA	Sheep and cattle density	PCA MR	Contamination index ranged from 8.3% to 41.3%. High soil contamination was not necessarily related to human incidence.
**Chen et al.** [65]	2014	Hubei, China	Longitudinal study	Human:1287–9778 beginning-end of study Cattle: 821–693 beginning-end of study Snail: 46078–15010 beginning-end of study	Schistosomiasis	IHA Miracidial hatching test Microscopy	Fecal-matter containers Lavatories Water supply	Cattle replacing with machinery Fencing of cattle Mollusciciding Chemotherapy	X^2^ Fisher’s exact test Spearman correlation	Prevalence in humans declined from 1.7% to 0.4% in 7 years. Prevalence in bovines decreased from 11.7% to 0.6% in 7 years.
**Dattoli et al.** [66]	2011	Salvador, Brazil	Cross-sectional	1217 children 4–11 years	Toxoplasmosis	ELISA	Flush toilet Water source Sewage system	Rodents, cats, dogs in the household	BR MR	Prevalence was 17.5%. Risk factors included presence of cats in the household, non-treated water pipes and absence of a flush toilet at home.
**Eshitera et al.** [67]	2012	Homa Bay, Kenya	Cross-sectional	392 pigs	Cysticercosis	ELISA Tongue	Latrine use	Pig housing	BR	Prevalence was 32.8%. Main identified risk factors was belonging to a household were latrine use was not evident. There was a predominance of free-ranging pigs.
**Fernandes et al.** [68]	2016	Paraiba, Brazil	Cross-sectional	1043 dogs	Leishmaniasis / Trypanosomiasis	IFAT	Housing condition Water dams	Contact with dogs, cattle, horses, cats, goats, sheep, pigs	UR MR	Prevalence of canine leishmaniasis was 7.8%, prevalence of Chagas Disease (CD) was 7.9%. Risk factors for CD were free housing of dogs and contact with bovines.
**Ganaba et al.** [69]	2011	Burkina Faso	Cross-sectional	888 pigs	Cysticercosis	ELISA	Latrine presence Water source	Farming system Livestock presence	UR MR	Prevalence ranged from 39.6% to 0%. Infection was not associated with lack of latrines, the source of drinking water or the status of infection in humans, but it was associated with free-roaming pigs during the rainy season.
**Holt et al.** [51]	2014	Luang Prabang-Savannakhet, Lao PDR	Cross-sectional	895 humans, 647 pigs	HEV/Trichinella spiralis/Cysticercosis/JEV	Serology	Toilet use Water source Water boiling	Pigs in household Pig handling Pig housing	MCA HCPC X^2^ MR	Results explained further in the next manuscript section.
**Hong et al.** [70]	2013	Hubei, China	Cluster randomized controlled trial	Human: 5323 control 5050 intervention Bovine: 313 control 318 intervention Snail: 9493 control 15490 intervention	Schistosomiasis	IHA Miracidial hatching test Microscopy	Fecal-matter containers Lavatories Water supply	Fencing of cattle Mollusciciding Chemotherapy	X^2^ Fisher’s exact test GLM Variance-covariance	Prevalence decreased from 3.41% to 0.81% in humans, 3.3% to 0% in bovine in a period of 3 years.
**Hunter et al.** [71]	2015	Hai, Tanzania	Case-control	218 human cases, 174 controls	Cysticercosis	WBA CT scan	Toilet type Water source	Keeping of pigs	X^2^ Fisher’s exact test	Prevalence for taeniasis was 2.8%. Sanitation and pig-keeping practices were not deemed risk factors for neurocysticercosis.
**Jayashi et al.** [72]	2012	Morropon, Peru	Cross-sectional	1153 pigs	Cysticercosis	EITB	Latrine presence	Free-roaming pigs	BR MR	Prevalence was 45.19%. Latrine presence acted as a protective factor. Rearing system did not represent a risk or a protective factor.
**Kagira et al.** [73]	2010	Busia, Kenya	Cross-sectional	221 pigs	Cysticercosis	ELISA	Latrine presence	Free-roaming pigs	X^2^ MR	Prevalence was 4%. Risk factor associated was lack of latrines at the household level.
**Kankya et al.** [74]	2010	Mubende, Uganda	Retrospective	253 individuals	Nontuberculous mycobacteria	Questionnaire	Water usage Water source Water storage Wild animal water source sharing	Wild animal presence Livestock managing	UR MR	Relevant risk factors identified were sharing of water sources between humans and animals, use of spring water instead of stream water, non-separation of water containers for drinking ad domestic use, cattle keeping and distance of household to animal night shelters of over 20 metres.
**Komba et al.** [75]	2013	Mbeya, Tanzania	Cross-sectional	600 pigs	Cysticercosis	ELISA	Latrine presence Latrine condition Water source Presence of faeces	Pig management system	MR	Prevalence was 32%. Risk factors include free roaming of pigs, previous porcine cysticercosis in the household and sourcing of water from rivers.
**Krecek et al.** [76]	2012	Eastern Cape, South Africa	Cross-sectional	261 pigs	Cysticercosis	ELISA	Latrine presence Water source	Pig husbandry system	BR MR	Prevalence was 57%. Main risk factor identified was the absence of latrines in the household.
**Lau et al.** [77]	2016	Fiji	Cross-sectional	2152 participants	Leptospirosis	MAT	Metered water	Presence of pigs Cattle density Animal presence and contact	UR MR	Prevalence was 19.4%. Risk factors included lack of treated water at home, pigs in the community and high cattle density.
**Luke et al.** [78]	2013	Kanese, Uganda	Cross-sectional	384 participants	Echinococcosis	Questionnaire	Hand washing Water boiling	Dog faeces disposal Close contact with dogs Grazing livestock with dogs	Percentage MR	Potential risk factors identified included dog ownership, presence of stray dogs, home slaughtering of animals, lack of hand washing and lack of water-boiling practices.
**Magalhaes et al.** [79]	2016	Fernando de Noronha Archipielago, Brazil	Cross-sectional	430 chickens	Toxoplasmosis	IFAT	Water source	Cat presence, domestic and feral	X^2^ Fisher’s exact test UR	Average prevalence was 88.4%. Risk factors included number of domestic cats in the properties, presence of feral cats and presence of an open water source.
**Mendoça et al.** [80]	2013	Sergipe, Brazil	Cross-sectional	932 sheep	Toxoplasmosis	IFAT	Water source	Cat presence	BR MR	Prevalence was 28.22%. Risk factors included presence of cats in the property. Consumption of water from the source or a deep well acted as protective factors.
**Miller et al.** [81]	2014	Kiruhura-Bushenyi, Uganda	Cross-sectional	236 humans, 768 cattle, 315 goats, 635 bovine milk samples	Brucellosis	Lateral flow assay Rose Bengal Milk ring test	Wildlife water sharing Water source	Wildlife presence/ contact/ housing Flock/herd density	X^2^ Fisher’s exact test MR	Prevalence was 14% in cattle serum, 29% in cattle milk, 17% in goat serum and 11% in human serum. Relevant risk factors identified include sharing of water source between farm and wild animals (lack of biosecurity) and free grazing.
**Mwang’onde et al.** [82]	2014	Mbulu, Tanzania	Cross-sectional	80 participants	Cysticercosis	Questionnaire	Toilet condition Toilet usage	Free-ranging pigs	UR	Risk factors identified include indiscriminate defecation, improper use of toilets, free-roaming pigs, unregulated slaughtering and inadequate meat inspection.
**Mwape et al.** [83]	2012	Petauke, Zambia	Cross-sectional	708 serum and 718 stool samples	Cysticercosis	ELISA PCR	Latrine presence	Pig husbandry	UR MR	Prevalence was 6.3%. Risk factors included free-range pig husbandry, and lack of latrines in the household.
**Ngowi et al.** [84]	2008	Mbulu, Tanzania	Intervention-trial	827 pig-keepers 827 piglets	Cysticercosis	ELISA	Hand washing Latrine use Latrine condition Water boiling	Free-ranging pigs	Poisson model Wilcoxon matched-pairs signed-rank test	Knowledge about transmission and prevention increased and incidence decreased to almost half in 10–12 months of educational intervention.
**Ngowi et al.** [85]	2004	Mbulu, Tanzania	Cross-sectional	770 pigs	Cysticercosis	Tongue	Latrine usage	Free-roaming pigs	Bayesian model	Prevalence was 17.4%. Risk factors included lack of latrines in the household. Prevalence in households without latrines was 14.5% given a rate of free-roaming of pigs of 96%.
**Ngwing et al.** [86]	2012	Bafut-Santa, Cameroon	Cross-sectional	499 pigs	Cysticercosis	Tongue ELISA	Toilet presence	Free-roaming pigs	Descriptive X^2^	Prevalence was 3.6% for tongue examination and 7.6% for ELISA. Risk factors included roaming of pigs, faecal disposal in the environment and poor sanitation.
**Nkouawa et al.** [87]	2015	Bangoua, Cameron	Cross-sectional	384 participants	Cysticercosis / Taeniasis	ELISA Immunoblot	Latrine presence Water source	Farming system Pig presence	Fisher’s exact test	Prevalence was 3.1%. Risk factor identified was consumption of pork meat after home slaughter. Penning of pigs and good hygiene practices rendered factors such as non-drinkable water as non-risky.
**Ogendi et al.** [88]	2013	Thika, Kenya	Cross-sectional	385 farmers	Toxoplasmosis	Questionnaire	Water boiling Water source	Cat housing Cat keeping	Percentage	Most households had good water and sanitation conditions. 44.9% owned cats; of those, only 2.8% had litter boxes and none used gloves for emptying them.
**Pinheiro et al.** [89]	2011	Minas Gerais, Brazil	Cross-sectional	2367 stool samples	Giardiasis	Microscopy	Water quality Water source Running water points Sanitary infrastructure Sewage discharge	Ownership of pets	X^2^ UR	Prevalence was 6.1%. Risk factors identified included inadequate sewage discharge, drinking of unsafe water and lack of sanitary infrastructure. Ownership of pets was not deemed a risk factor.
**Pouedet et al.** [90]	2002	Bafou-Bamendou, Cameroon	Cross-sectional	707 pigs	Cysticercosis	ELISA Tongue	Latrine presence	Free-roaming pigs	Bayesian model Z-test	Prevalence was 10.9%. Risk factors included free-roaming of pigs and access of pigs to human faeces. Presence of latrines was not associated with risk of infection.
**Prasad et al.** [91]	2007	Uttar Pradesh, India	Cross-sectional	924 human subjects	Taeniasis	Microscopy	Hand washing Garbage disposal	NA	UR MR	Prevalence was 18.6%. Risk factors included poor hand hygiene.
**Prasad et al.** [92]	2011	Uttar Pradesh, India	Cross-sectional	595 humans	Neurocysticercosis	MRI EITB	Water source Water drainage	Pig housing	UR	Prevalence was 15.1%. Risk factors included lack of safe drinking water, inadequate drainage system and not keeping pigs separate from the household.
**Pray et al.** [93]	2016	Piura, Peru	Cross-sectional Longitudinal	37 pigs	Cysticercosis	GPS tracking Questionnaire	Latrine presence Latrine usage	Pig interaction with defecation areas	Localized Convex Hulls	The average pig’s roaming area with risk of interaction with human faeces was calculated at 100m.
**Rebecca et al.** [94]	2012	Jos, Nigeria	Cross-sectional	125 pig rearers	Taenia solium	ELISA	Toilet presence Toilet condition Hand washing	Pig management system	X^2^ Fisher’s exact test	Prevalence was 9.6%. Risk factors included open defecation, lack of hand washing after defecating and extensive rearing of pigs.
**Rossi et al.** [95]	2015	Sao Paulo, Brazil	Cross-sectional	190903 bovines	Cysticercosis	Retrospective diagnosis	Water source	Fishing activities	UR MR	Prevalence was 2.26%. Risk factors included access of cattle to a non-controlled water source and sport fishing activities near the farms.
**Sarti et al.** [96]	1992	Michoacan, Mexico	Cross-sectional	216 pigs	Cysticercosis	Tongue	Latrine presence	Pigs access to garbage/faeces	X^2^ Fisher’s exact test	Prevalence was 6.5%. Risk factors included access to human faeces for pigs, presence of an indoor latrine and the indiscriminate disposal of human faeces around the household.
**Sato et al.** [97]	2006	Piracuruca, Brazil	Cross-sectional	7 human blood samples	Cysticercosis	ELISA PCR	Water source	NA	NA	The study identified a relationship between cysticercosis endemicity and extensive pig farming and lack of water treatment.
**Schantz et al.** [98]	2003	Qinghai, China	Cross-sectional	3703 volunteers	Echinococcosis	ELISA	Hygienic practices Water source	Animal ownership Dog presence	UR MR	Prevalence was 6.6%. Livestock ownership and dog presence indoors were significant risk factors, as well as consuming untreated water.
**Sikasunge et al.** [99]	2007	Petauke-Katete, Zambia	Cross-sectional	384 pigs	Cysticercosis	ELISA Tongue	Presence of latrine	Husbandry system	MR	Prevalence was 12.7–32.1% for tongue examination and 30–51.7%. The significant risk factor identified was free-roaming of pigs. Lack of latrines was deemed non-significant as a risk factor.
**Sikasunge et al.** [100]	2008	Petauke-Katete-Gwembe-Monze-Mongu, Zambia	Cross-sectional	1691 pigs	Cysticercosis	ELISA Tongue	Latrine presence	Free-ranging pigs	BR	Prevalence of tongue examination was 10.8%, ELISA prevalence being 23.3%. Latrine presence and free-roaming pigs were not found significant as risk factors.
**Sun et al.** [101]	2011	Jiangsu, China	Longitudinal	Human:140868–252323 Cattle: 5424–1604 Snail: 585298–295384 beginning-end of study	Schistosomiasis	DDIA Miracidial hatching test Microscopy	Fecal-matter containers Lavatories Water supply	Cattle replacing Fencing of cattle Mollusciciding Chemotherapy	X^2^	Intervention reduced prevalence to 0% in a period of 3 years.
**Sun et al.** [102]	2015	Heilongjiang-Liaoning-Shandong-Hebei, China	Cross-sectional	4487 bovine blood samples	Toxoplasma gondii, Neospora caninum, Chlamydia abortus, bovine viral diarrhoea virus	Serology	Hygiene practices Water source	Management system Presence of sheep, goats, pigs, rodents, poultry, cats, dogs	BR MR	Prevalence was 27.16% for *T*.*gondii*, with unprotected water source and presence of felids close to the herd.
**Thys et al.** [103]	2015	Petauke, Zambia	Cross-sectional	172 participants	Cysticercosis	Questionnaire	Latrine use	NA	Descriptive	Latrines were not constructed in the household due to availability of communal latrines. Men were reluctant to stop open defecation due to cultural taboos.
**Tilahun et al.** [104]	2015	East Hararghe Zone, Ethiopia	Cross-sectional	354 participants	Toxoplasmosis	Serology	Water source	Cats presence Feral cat presence	UR MR	Prevalence was 65.8% for IgG and 8.98% for IgM. Risk factors included pipe water source and keeping cats at home.
**Tsegay et al.** [105]	2016	Ethiopia	Cross-sectional	418 cart horses	Leptospirosis	MAT	Water source	Presence of domestic animals Presence of rodents	UR MR	Prevalence varied from 5.3% to 62.1%. Risk factors included drinking river water and presence of dogs in neighbouring properties.
**Wang, Chen et al.** [106]	2009	Jiangxi, China	Intervention control trial	Human:300–375 Snail: 1054–1171 beginning-end of study	Schistosomiasis	Microscopy	Fecal-matter containers Lavatories Water supply	Cattle replacing with machinery Fencing of cattle Mollusciciding Chemotherapy	X^2^	Intervention reduced rate of infection from 11.3% to 0.7% and from 4.0% to 0.9% in each village.
**Wardrop et al.** [107]	2015	Kenya	Cross-sectional	2113 humans, 93 pigs	Cysticercosis	ELISA Microscopy	Latrine use Latrine presence Latrine type Water source	Pig keeping	UR MR	Prevalence was 6.6% in humans and 17.2% in pigs. The significant risk factor identified was the use of well water for drinking.
**Widdowson et al.** [108]	2000	Yucatan Peninsula, Mexico	Cross-sectional	697 pigs	Cysticercosis	Immunoblot assay	Toilet presence Water source	Pig husbandry	UR MR	Prevalence was 29%. Presence of toilet was found to be a risk factor as opposed to absence of one. Both corralling and non-corralling of pigs were found to be equally impactful risk factors.
**Wohlgemut et al.** [109]	2010	Busia, Kenya	Educational intervention	282 farmers	Cysticercosis	Questionnaire	Latrine use	Pig husbandry	X^2^ MR	Knowledge of transmission and penning of pigs improved after the first and second educational workshops.
**Yang et al.** [110]	2009	Sichuan,China	Cross-sectional comparative	580 dogs, 100 yaks, 15 goat, 19 sheep	Echinococcosis	ELISA Necropsy	NA	Dog treatment	X^2^	Prevalence of echinococcosis decreased amongst the dog population after 5 years of treatment. The intervention had no positive impact in the potential for re-infection.
**Yohana et al.** [111]	2013	Iringa, Tanzania	Cross-sectional	308 pigs	Cysticercosis / Taeniasis	Tongue	Water source Water boiling Toilet presence	Pig husbandry	UR X^2^	Prevalence was 7.5%. Risk factors included lack of access to tap water, lack of toilets and free ranging of pigs.
**Zhang et al.** [112]	2015	China	Cross-sectional	1842 participants	Toxoplasmosis	ELISA	Water source Hygiene practices	Cat in household Dog in household	BR MR	Prevalence was 13.79% for IgG and 1.25% for IgM. Risk factors included well/river water source and cat presence in the household.
**Zirintunda et al.** [113]	2014	Soroti, Uganda	Cross-sectional	25 transects	Cysticercosis	Observational	Latrine presence Latrine use	NA	Descriptive	Despite a latrine coverage of 46%, human faeces were seen around houses and latrines, in a community with a majority of pigs farmed extensively.

brpca: Chi-square; MR: Multivariate Regression; UR: Univariate Regression; BR: Bivariate Regression; PCA: Principal Component Analysis; MCA: Multiple Correspondence Analysis; HCPC: Hierarchical Clustering on Principal Components; GLM: Generalised Linear Model.

**Fig 2:**
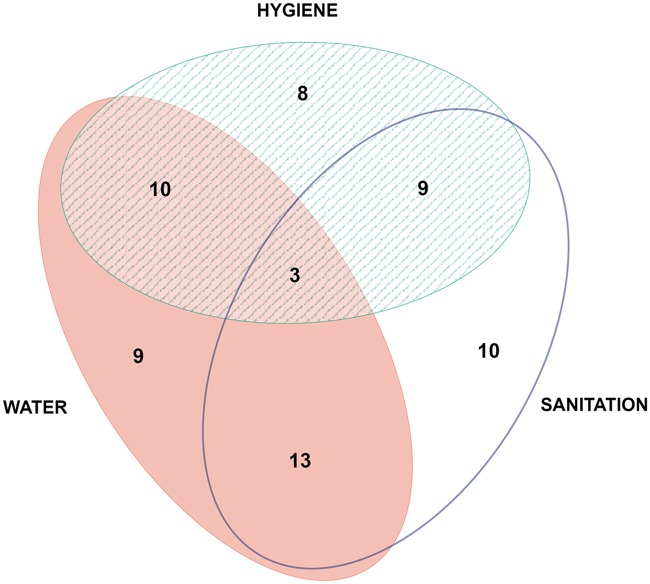
Number and type of WASH intervention categories studied simultaneously by the articles.

More than half of the studies (29) focused on cysticercosis, while 12 focused on toxoplasmosis (Table 2). Humans appear as the most studied species, with 36 studies looking at human burden of disease, while pigs were second with 26 citations. Fifty one out of 64 were designed as cross-sectional studies, 46 of these establishing a prevalence value through a serological test and combining it with a questionnaire for associated risk factors. Table 3 shows the study count for each of the categories for water, hygiene and sanitation components, and the proportion of studies that included one, two, or the three types is shown in Fig 2. Three studies had at least one factor in each of the categories.

**Table 2:** Number of articles identified by disease and species studied.

Pathogen/Disease	Total Studies	Humans	Pig	Large ruminant	Small ruminant	Chicken	Turkey	Dog	Cat	Horse	Snail
**Cysticercosis**	29	11	21	1	-	-	-	-	-	-	-
**Toxoplasmosis**	12	6	-	2	3	1	-	-	-	-	-
**Schistosomiasis**	4	4	-	3	-	-	-	-	-	-	4
**Taeniasis**	4	3	1	-	-	-	-	-	-	-	-
**Echinococcosis**	4	2	-	1	1	-	-	2	-	-	-
**Leptospirosis**	3	2	-	--	-	-	-	-	-	1	-
**Giardiasis**	1	1	-	-	-	-	-	-	-	-	-
**Toxocariasis**	1	1	1	-	-	-	-	-	-	-	-
**Brucellosis**	1	1	-	1	1	-	-	-	-	-	-
**Helminthiases**	1	1	-	-	-	-	-	-	-	-	-
**Bovine tuberculosis**	1	-	-	1	1	-	-	-	-	-	-
**Leishmaniasis**	1	-	-	-	-	-	-	1	-	-	-
**HEV**	1	1	1	-	-	-	-	-	-	-	-
**JEV**	1	1	1	-	-	-	-	-	-	-	-
**Trichinosis**	1	1	1	-	-	-	-	-	-	-	-
**Mycobacteria NT**	1	1	-	-	-	-	-	-	-	-	-
**TOTAL**		36	26	9	6	1	0	3	0	1	4

**Table 3:** Number of articles by type of WASH factors studied.

Sanitation Hygiene component	Studies
*Type of water source*	29
*Latrine/toilet presence in the village/household*	22
*Latrine/toilet use (behavioural practice)*	13
*Faeces/waste disposal (behavioural practice)*	7
*Water for consumption quality*	7
*Hygienic conditions of the household*	6
*Hand washing (behavioural practice)*	5
*Water boiling (behavioural practice)*	5
*Other hygienic practices (behavioural practice)*	4
*Latrine/toilet condition*	4
*Type of water drainage*	3
*Latrine/toilet type in the village/household*	2
*Hygiene knowledge*	1

The summarised data suggests the existence of a relationship between NZD epidemiology and the contact of humans and animals in the household, generally showing a negative impact of animal presence on WASH measures or an enhanced negative effect of animal presence on the impact of poor WASH conditions. In the case of cysticercosis, studies show contradictory results regarding the impact of WASH measures and animal presence on disease prevalence.

### Final review

Due to the small number of studies that were selected based on the criteria, the outcome of the quality control check was not considered for further exclusion.

The study by Holt et al. (2016) was designed as a cross-sectional study examining prevalence of hepatitis E virus (HEV), Japanese encephalitis virus and Trichinella spiralis in both humans and pigs, as well as Taenia spp. solely in humans in two provinces of Lao PDR, with a multiple correspondence analysis and a hierarchical clustering of several components deemed relevant to disease transmission. Three clusters were identified: one referential (*cluster 1*) with the best sanitation and lowest pig contact; *cluster 2*, with moderate sanitation levels and slaughtering of pigs as the main source of animal contact; and *cluster 3*, with lower sanitation levels and a relative higher rate of free-roaming pigs. The risk of human infection, measured through Odds Ratio (OR), for each of the diseases and clusters when compared to cluster 1 are shown in Table 4. HEV had a very similar OR for risk of infection between clusters 2 and 3, despite the superior WASH conditions of cluster 2. For Taenia spp. and Cysticercosis, risk of infection proved higher in cluster 3 than cluster 2, but with a significant increased risk of infection in cluster 2 compared to the control, despite solid practices of hand washing and water boiling amongst the population. Finally, Japanese encephalitis showed an increased risk of infection in cluster 2 over cluster 3, despite better WASH conditions. Data regarding pig seropositivity was not clustered and WASH factors were not found to be significant in T. spiralis and HEV infection.

**Table 4:** Variables and Odds-Ratio for the diseases studied. Source: Holt et al, 2016 (page 11).

Disease	OR (95% Confidence Interval)
*HEV*	
Cluster 1 (Better sanitation, lower pig contact)	1
Cluster 2 (Moderate sanitation, higher direct pig contact)	2.18 (1.37 to 3.45)
Cluster 3 (Poorer sanitation, higher indirect pig contact)	2.30 (1.58 to 3.33)
*T*. *spiralis*	
Cluster 1 (Better sanitation, lower pig contact)	1
Cluster 2 (Moderate sanitation, higher direct pig contact)	0.52 (0.33 to 0.82)
Cluster 3 (Poorer sanitation, higher indirect pig contact)	0.42 (0.28 to 0.61)
*Taenia spp*.	
Cluster 1 (Better sanitation, lower pig contact)	1
Cluster 2 (Moderate sanitation, higher direct pig contact)	2.76 (0.78 to 9.72)
Cluster 3 (Poorer sanitation, higher indirect pig contact)	3.38 (1.12 to 10.2)
*Cysticercosis*	
Cluster 1 (Better sanitation, lower pig contact)	1
Cluster 2 (Moderate sanitation, higher direct pig contact)	1.85 (0.55 to 6.23)
Cluster 3 (Poorer sanitation, higher indirect pig contact)	2.69 (1.12 to 10.2)
*JEV*	
Cluster 1 (Better sanitation, lower pig contact)	1
Cluster 2 (Moderate sanitation, higher direct pig contact)	2.49 (1.12 to 5.19)
Cluster 3 (Poorer sanitation, higher indirect pig contact)	1.18 (0.54 to 2.52)

The other study (Bulaya et al. 2015) was a comparative study pre- and post- community-led total sanitation (CLTS) intervention for porcine cysticercosis control, identifying prevalence performing an Ag-ELISA test. There was no randomization in village selection or house selection, and instead selected based on village characteristics and willingness to participate, respectively. The prevalence pre-intervention was 13.5%, (6.8–20.1, 95% C.I.), compared to a value of 16.4% (12–20.8, 95% C.I.) post-intervention, although this increase was deemed non-significant by the author. After the intervention, latrine presence improved from 67.2% to 83.1%, with the percentage of free-roaming pigs changing from an 89.8% to a 30.3% of them free roaming, 43.8% partially free roaming and 25.8% penned. Home slaughter of pigs increased from 49.15% baseline to 80.90% post-intervention. Despite the improvement in latrine presence, animal husbandry was not improved enough to avoid direct and indirect contact between animals and humans within the household.

## Discussion

This review showed examples of the way animal-human interaction can affect the effectiveness of WASH interventions for zoonosis control. Importantly, it also highlighted the dearth of studies looking specifically at this interaction. After the search retrieved 7588 articles for this review, 64 were selected in the first screening, of which only 2 were selected for the final review after the second screening. This outcome is likely due to the sectoral focus of the studies. Traditionally, research groups investigating the effectiveness of WASH interventions focus on human factors as positive or negative influences. Similarly, the Veterinary Public Health community focuses more on animal-related factors and disease-transmission routes. The interaction between these two aspects is a research and programming ‘blind spot’, as was demonstrated by this review, and needs to be addressed with further intersectoral research studies.

As noted by Zinsstag in 2015 [33], a study in Vietnam showed how a One Health approach for WASH programmes integrates all factors into one framework. This helps identify the relationship between the factors, while exposing the missing links and the areas in need for further research, of which the main one stated is “the boundaries of the sanitation problem”. Sanitation and hygiene programmes have proven effective in reducing NTD burden in numerous studies, as backed by various systematic reviews [43–45]. However, effective, full-coverage implementation of control programmes considering both human and animal sanitation aspects can be challenging in practice. As described by Guilman et al. in 2012 [26], some communities may not have sufficient resources to change their animal farming system to one that limits animal-human contact. In other cases, the community may actually benefit economically from this new farming system [114], but as long as the population believes this is not the case, no change will be embraced by the community [115]. This reinforces the importance of accompanying these type of logistic measures with strong education and hygiene promotion campaigns that involve the community and show the importance and benefits of adopting them.

The study by Holt et al. [51] compared Odds Ratio of infection in several pig zoonoses between different sanitation and pig contact factors. For HEV, lower levels of sanitation, as described in the results section, proved to be a risk factor for virus presence, without significant differences between these lower levels specifically. However, increased contact with pigs, particularly through handling and slaughtering, proved significant in its influence on the effectiveness of WASH measures in disease control, as the cluster with moderate sanitation and close pig contact had equal risk of infection as the cluster with poorer sanitation. Pig contact has been described as a risk factor for HEV transmission previously [116], but according to this study, pig corralling impede their access to the household would not make a significant difference in disease transmission as long as the animals are still being slaughtered at home, due to direct human contact with pig blood. In the case of *Trichinella*, socioeconomic status acted as a confounder, since the main risk factor is pork consumption [117, 118], which in this study was associated with higher status due to availability and affordability cost, as are good sanitation and hygiene conditions. In the case of JEV, the cluster with higher direct contact with pigs showed a higher risk of infection, despite better sanitation and hygiene conditions, showing an example of how animal contact can severely hinder the effectiveness of WASH measures. This could be due to its vector-borne nature, which correlates to two factors of this particular cluster: unprotected water sources, which facilitates breeding areas for *Culex spp*.; hygiene practices, latrine use or corralling measures would not make a significant impact in its transmission unless done optimally, avoiding contamination of water that could facilitate *Culex spp*. reproduction. Regarding *Taenia solium* and cysticercosis, the cluster with higher rates of free-roaming pigs and open defecation showed the highest risk of infection, as expected. However, the high risk of infection presented by the cluster with moderate WASH and close contact with pigs shows how the latter can affect the effectiveness of the former.

During the selection process of this review, several studies (Table 1) were screened and later revisited, for further insights on the impact of animals on WASH interventions. Some showed presence, usage or condition of latrines and free roaming of pigs to be significant risk factors in disease transmission [84, 119, 120], but others had non-significant results [107], rather identifying the source of water for consumption and its quality as a risk factor. In contrast, Nkouawa et al. in 2015 [87] identified that despite having a non-potable (unsafe) water source, disease transmission was reduced by improving hygienic practices and corralling pigs. The study by Holt et al. [51] provided robust results on relative impact of animal and WASH factors, meeting the criteria for selection stated in the protocol of the review. However, future studies should ideally be designed in a way that focuses on isolating the influence of animal factors on the effectiveness of WASH measures. This is particularly difficult to achieve given the circumstances of the communities in which these studies need to be conducted: as noted by Schmidt et al. in 2014 [121], designing impact studies on water, sanitation and hygiene and retrieving significant results is a recurrent challenge for the scientific community: Randomised controlled trials are rarely free from bias, while observational studies usually lack a large enough study population or result significance [121]. Additionally, performing randomised controlled trials in the optimal representative geographical areas is logistically and economically challenging. Another factor to take into account is time, since marketing and promotion campaigns can take several years to have a significant effect, deeming any study that withholds investment in WASH services for such an extended period of time unethical [121].

A relevant limiting factor to assess the efficiency of any WASH programme implementation is the correct use, design and upkeep of sanitation facilities. Several studies show that although latrines were present in the community, they were not consistently used for defecation by all household members or kept in a sufficiently hygienic state [84, 85]. The incorrect use of latrines is often associated with socio-cultural and psychological factors, as identified by Thys in 2015 [122], such as a sense of reduced privacy, latrines being too close to the village, comfort of use or trust in its efficacy and need of use. Lack of ownership of the need for latrine construction and lack of ongoing support for maintenance and improvement can undermine potential health benefits of basic latrines.

The study by Bulaya et al. in 2015 [50], showed that despite the CLTS intervention resulting in increased latrine presence, net increase in latrine usage and improved pig husbandry, prevalence of disease in pigs increased slightly after the intervention. The study did not specify whether the newly built latrines resulted in safe separation of humans and animals from human faeces. Achieving that level of detail in the analysis is an objective for future studies. Although deemed non-significant, the 95% C.I. shows almost no change in prevalence from pre to post intervention. This was attributed by the authors to infected members of the community still practising open defecation due to lack of resources for latrine construction. Not corralling the totality of the pig population, therefore allowing for interaction of animals and humans within the household, could be the explanation as to why the increase in latrine presence had no effect in decreasing porcine cysticercosis. Free roaming of pigs has been identified as a risk factor for porcine cysticercosis by some of the studies screened before review inclusion [69, 75] but was found to be non-significantly others [72]. Similarly, the presence of latrines can be significant [72, 73] or non-significant [69] for disease prevalence in pigs, depending on the study, reinforcing the findings by Bulaya et al. (2015). As previously mentioned, low latrine usage has been described as a risk factor for disease transmission [59, 84, 85] but also as a recurrent sociocultural problem, since many members of the community do not use latrines on a consistent basis for a variety of reasons [59, 115, 122], or do not keep the latrines in a suitable condition for them to effectively reduce disease transmission [84, 115, 120]. However, poor programme design, lack of follow up or disputes between NGOs and community leaders on logistics, provisions and payments can be a cause for poor latrine construction and maintenance [123]. This reinforces the suggestion made by Bulaya et al.[50] of the importance of continued hygiene promotion programmes and access to sanitation hardware options in order to ensure the complete effectiveness of sanitation or animal husbandry improvement programmes.

As an example of a multifactorial approach to disease transmission control, prevalence of Schistosomiasis was significantly reduced in three studies in China [70, 102, 124] by implementing a complete WASH programme with sanitation facilities and hygiene educational programmes, reducing the indirect contact of animals and humans through water and reducing the population of the host snail species for *Schistosoma*. However, programmes that alter animal husbandry in drastic ways such as changing free-roaming farming systems into stabling farming systems, also alter the local economy of the community [125]. In the case of cysticercosis, the penning of pigs is not always possible in certain communities given the resulting increased costs of feed and infrastructure [125]. Substantial investment and economic compensation to farmers and households would therefore be required to maintain and sustain these programmes consistently over time [126].

In the case of toxoplasmosis, principal and consistent risk factors for infection identified throughout the literature, include unsafe water source, inadequate hygienic conditions of the household and cat presence in the household or the vicinity, and were common to human [52, 66] or animal [55, 58] infection. While providing clean water sources and creating appropriate hygienic conditions decreases the burden of disease, avoiding the presence of cats within the household could potentially increase the presence of rodents in many communities that use cats as the sole method of rodent control. A study showed how, when combined, the presence of cats and dogs in an area significantly reduced the local rodent population [127], however, more research should be conducted to clarify the impact of cat population control on rodent-transmitted diseases in rural communities.

The review protocol was designed to include animal-focused studies as well as human-focused studies to ensure a One Health approach to zoonotic disease transmission. Particularly for NZDs, interrupting sustained transmission requires a multifactorial approach considering both zoonotic and anthroponotic transmission paths. Reducing animal burden of disease has a direct effect on human prevalence of disease and vice versa [128], and therefore WASH programmes applied equally to human and animal populations are likely to provide better results than a human-centred approach. The review identified the lack of studies looking at the importance of animal influence in WASH programmes, exposing the existent lack of knowledge in the matter. Further research and programme design need to focus further on animal impact and isolating the study of animal components in the efficiency of WASH control programmes. One of the limitations of the review was the non-inclusion of rodent species in the study. Although rodents are acknowledged to be a source of NZD transmission within the household, they were deemed to overreach the scope and feasibility of this review: on one hand because the review focused in farmed animals kept by the household owners; on the other hand because thorough control of rodent activity in the household is difficult and less reliable than that of farmed animals, mainly due to the complex biological and ecological characteristics of each local rodent species [129, 130]. The initial literature review was conducted for fulfilment of an MSc with one student. All three co-authors advised on the approach to be taken and made revisions to the literature. Throughout the writing of the literature there was input from all authors who also held regular review meetings. To further optimise the systematic review, a second reviewer would have performed the search and selection and compared results. Also, had a longer period of time been available, more databases could have been screened, although the final count of studies would most likely be low, since the tendency identified in the review is that of a very low percentage of studies looking specifically at animal influence in WASH measures efficacy. The time constraints were due to the timelines of the MSc. However, all authors had additional input to the manuscript. Whilst the initial literature review was conducted by one student, the manuscript has been prepared after revisions by all authors with additional literature added after further reviews. This has been rewritten to reflect the input following the initial MSc project.

## Conclusions

This systematic review demonstrated the relevance of human-animal interaction within the household for the effectiveness of WASH measures for control of NZDs. It also shows the significant lack of specific studies tending to the effect of animals on WASH programmes’ effectiveness for zoonotic disease control. Several examples exist in the literature describing prevalence of zoonotic disease and associated risk factors, yet, in the majority of cases, their design fails to assess the specific influence of animal presence in WASH interventions. Further research should be undertaken regarding the influence of animals in WASH programmes, ideally isolating the sanitation component and studying different levels of animal interaction and exposure within the household. Attention to animal burden together with human burden of disease would allow for better understanding and optimisation of WASH programme effectiveness on both disease control and broader development objectives. There exists an evident lack of direct coordination between WHO’s WASH and NTDs official programmes. Further developing of a research agenda around the animal-sanitation-disease link can help set out clear actions on which disease control programmes can be based.

## Supporting information

S1 File Systematic review protocol. (DOCX)

S1 Checklist PRISMA checklist. (DOC)

S2 Checklist PRISMA flow diagram. (DOC)
