# Supplementary material for: Animal influence on water, sanitation and hygiene measures for zoonosis control at the household level: A systematic literature review
Source: PLoS Negl Trop Dis. 2018 Jul 12;12(7):e0006619. doi: 10.1371/journal.pntd.0006619 (PMC6057674; doi:10.1371/journal.pntd.0006619)
Supplement: S1 File — (DOCX) [file pntd.0006619.s001.docx]

**S1 File: Systematic review protocol**

- **Objectives**
  - Identify linkages between household animals and water, sanitation and hygiene interventions for zoonotic disease control
  - Identify knowledge gaps in the literature
- **Inclusion criteria**
  - **Types of studies**
    - Randomised control trials
    - Control trials
    - Quasi-experimental trials
    - Cohort studies
    - Case-control studies
    - Cross-sectional studies
    - Case-report studies

Only published studies will be considered for this review. The existent lack of information available regarding animal presence in the household and sanitation program-effectiveness requires the researching team to include all the types of study mentioned above in the review, despite their strength of evidence, as long as they meet the inclusion criteria.

- - **Types of participants**

The study will focus on human population in rural communities of low and middle income countries in close contact with animals, mainly those cases where animals are present in the household but also those in which animals are kept close to the house and the water, sanitation and human/household waste disposal facilities.

- - **Types of interventions**
    - Community led total sanitation (CLTS)
    - Sanitation marketing
    - Latrine construction
    - Household Water Treatment and Safe Storage (HWTS)
    - Improved waste disposal (animal and human)
    - Improved faeces disposal (animal and human)
    - Fresh water supply
    - Hygiene promotion interventions
  - **Types of outcomes**
    - Primary
      - Human burden of disease
        - Morbidity (incidence and prevalence)
        - Mortality
        - DALYs
        - QALYs
      - Animal burden of disease
        - Morbidity (incidence and prevalence)
        - Mortality
        - Production losses
    - Secondary
      - Risk factors
      - Knowledge gaps
      - Adverse effects
      - Hygiene promotion programme acceptance/efficacy
- **Methods**
  - **Search strategy**
    - Databases
      - Medline
      - Web of Science
      - Global Health
    - Search terms:

Four pools of terms regarding water, sanitation and hygiene, animals, diseases and location will be combined, with at least one term from each pool. Terms with an asterisk next to them indicate that derivatives of the word will also be used for searching. Terms within columns will be combined with the Boolean “OR”; columns will be combined with the Boolean “AND”.

| **Sanitation and hygiene** | **Animals** | **Disease** | **Location** |
| --- | --- | --- | --- |
| Borehole | Bovine | Taeniasis | Albania |
| WASH | Buffalo | Zoonosis | Algeria |
| Education | Cat | Cysticercosis | American Samoa |
| Toilet | Chicken | Neglected zoonotic disease | Angola |
| Pit | Corralling | Nagana | Azerbaijan |
| Water supply | Cow | Neglected tropical disease | Belarus |
| Water treatment | Dog | Taenia solium | Belize |
| Open defecation | Farming | Echinococc* | Bosnia Herzegovina |
| CLTS | Goat | Zoonotic | Botswana |
| Sanitation | Horse | NTDS | Brazil |
| Standpipe | Ovine | Pig tapeworm | Bulgaria |
| Latrine | Pig | NZD | China |
| Rainwater | Porcine | Trypanosom* | Colombia |
| Sanitary engineering | Poultry | HAT | Costa Rica |
| HWT | Sheep | NZDS | Cuba |
| Water | Turkey | NTD | Dominica |
| Open urination |  | Hydatid* | Dominican Republic |
| Shower laundry |  | Schistosom* | Ecuador |
| Hygiene |  | Snail fever | Fiji |
| Detergent |  | Foodborne trematod* | Gabon |
| Soap |  | FBT | Grenada |
| Risk factor |  | Chlonorch* | Iran |
| Excre* |  | Fasciol* | Iraq |
| Faec* |  | Distomatosis | Jamaica |
| Fecal |  | Liver rot | Jordan |
| Feces |  | Opisthorch* | Kazakhstan |
| Hand washing |  | Paragonim* | Lebanon |
| Handwashing |  | Lung fluke | Libya |
| Waste management |  | Toxoplasm* | Macedonia |
| Waste disposal |  | Cryptosporid* | Malaysia |
|  |  | Crypto | Maldives |
|  |  | Brucell* | Marshall Islands |
|  |  | Anthrax | Mauritius |
|  |  | Anthracis | Mexico |
|  |  | Leptospir* | Mongolia |
|  |  | Shigell* | Montenegro |
|  |  | Escherichia coli | Namibia |
|  |  | Mycobacterium bovis | Palau |
|  |  | M. bovis | Panama |
|  |  |  | Paraguay |
|  |  |  | Peru |
|  |  |  | Romania |
|  |  |  | Serbia |
|  |  |  | South Africa |
|  |  |  | St. Lucia |
|  |  |  | St.Vincent Grenadines |
|  |  |  | Suriname |
|  |  |  | Thailand |
|  |  |  | Tonga |
|  |  |  | Tunisia |
|  |  |  | Turkey |
|  |  |  | Turkmenistan |
|  |  |  | Tuvalu |
|  |  |  | Armenia |
|  |  |  | Bangladesh |
|  |  |  | Bhutan |
|  |  |  | Bolivia |
|  |  |  | Cabo Verde |
|  |  |  | Cameroon |
|  |  |  | Congo |
|  |  |  | Cote D’ivoire |
|  |  |  | Djibouti |
|  |  |  | Egypt |
|  |  |  | El Salvador |
|  |  |  | Georgia |
|  |  |  | Ghana |
|  |  |  | Guatemala |
|  |  |  | Guyana |
|  |  |  | Honduras |
|  |  |  | India |
|  |  |  | Indonesia |
|  |  |  | Kenya |
|  |  |  | Kiribati |
|  |  |  | Kosovo |
|  |  |  | Kyrgyz |
|  |  |  | Lao |
|  |  |  | Lesotho |
|  |  |  | Mauritania |
|  |  |  | Micronesia |
|  |  |  | Moldova |
|  |  |  | Morocco |
|  |  |  | Myanmar |
|  |  |  | Nicaragua |
|  |  |  | Nigeria |
|  |  |  | Pakistan |
|  |  |  | Papua New Guinea |
|  |  |  | Philippines |
|  |  |  | Samoa |
|  |  |  | Sao Tome E Principe |
|  |  |  | Senegal |
|  |  |  | Solomon Islands |
|  |  |  | Sri Lanka |
|  |  |  | Sudan |
|  |  |  | Swaziland |
|  |  |  | Syria |
|  |  |  | Tajikistan |
|  |  |  | Timor |
|  |  |  | Ukraine |
|  |  |  | Uzbekistan |
|  |  |  | Vanuatu |
|  |  |  | Vietnam |
|  |  |  | Gaza |
|  |  |  | Yemen |
|  |  |  | Zambia |
|  |  |  | Afghanistan |
|  |  |  | Benin |
|  |  |  | Burkina Faso |
|  |  |  | Burundi |
|  |  |  | Cambodia |
|  |  |  | Central African Republic |
|  |  |  | Chad |
|  |  |  | Comoros |
|  |  |  | Eritrea |
|  |  |  | Ethiopia |
|  |  |  | Gambia |
|  |  |  | Guinea |
|  |  |  | Haiti |
|  |  |  | Liberia |
|  |  |  | Madagascar |
|  |  |  | Malawi |
|  |  |  | Mali |
|  |  |  | Mozambique |
|  |  |  | Nepal |
|  |  |  | Niger |
|  |  |  | Rwanda |
|  |  |  | Sierra Leone |
|  |  |  | Somalia |
|  |  |  | South Sudan |
|  |  |  | Tanzania |
|  |  |  | Togo |
|  |  |  | Uganda |
|  |  |  | Zimbabwe |

- - - Language
      - English
      - Spanish
    - Time
      - From 1980 to April 2016 (included) for each database
  - **Study selection**
    - Titles and abstracts of studies will be screened for relevance to the topic. Those studies considered not to be relevant will be excluded. Studies involving the topic, but perhaps not relevant on the grounds of population will be evaluated for further consideration.
    - Full text will be sought for all studies meeting the inclusion criteria. Final selection will be made by the reviewer. A flow chart will be provided for easier understanding of the process followed.
      - Screening of titles and abstracts
        - Out

Non-zoonotic disease

Language not English or Spanish

Full text not available

Article is a review

- - - - - In

Zoonotic disease

Sanitation and/or hygiene programme mentioned

Language is English or Spanish

Full text available

- - - - Study reading
        - Out

Animal influence not taken into account in the study

Study does not show human/animal data regarding burden of disease

Quality of the study is not deemed sufficient to be included in the review

- - - - - In

Study takes into account animals to evaluate effectiveness of water/sanitation/hygiene intervention/programme

Quality and bias-reduction is seemed appropriate to include the study in the review

- - **Data extraction**
    - Data will be extracted using a predesigned data extraction form. This will be a simple text based form in Word. The data will be entered on to the form to facilitate summarisation and writing of the final report. Sample data extraction form:

| **General** | **Study** | **Participants** | **Intervention/factors** | **Outcome** |
| --- | --- | --- | --- | --- |
| Date of extraction:  Author:  Title:  Citation:  Type of publication:  Country:  Funding: | Aim/Objectives:  Study design:  Inclusion criteria:  Exclusion criteria:  Recruitment type: | Number:  Age:  Ethnicity:  Animal species:  Disease:  Co-morbidities: | Main intervention:  Secondary intervention(s):  Animal factors  Human factors | Primary outcome:  Human burden  Animal burden  Secondary outcome(s):  Learning/correct usage  Knowledge gaps  Risk factors |

- - **Quality assessment**
    - Quality assessment will be performed following the guidelines for public health from the National Institute for Health and Clinical Excellence (NICE 2006).
  - **Data synthesis**
    - Due to the inclusion of various types of studies analysing different diseases and potential outcomes, pooling may not be possible. Due to lack of consistent information and time, synthesis will be performed narratively, grouping studies of the same type where appropriate.
