# Supplementary material for: Animal influence on water, sanitation and hygiene measures for zoonosis control at the household level: A systematic literature review
Source: PLoS Negl Trop Dis. 2018 Jul 12;12(7):e0006619. doi: 10.1371/journal.pntd.0006619 (PMC6057674; doi:10.1371/journal.pntd.0006619)
Supplement: S1 Checklist — (DOC) [file pntd.0006619.s002.doc]

| **Section/topic** | **#** | **Checklist item** | **Reported on page #** |
| --- | --- | --- | --- |
| **TITLE** | | |  |
| Title | 1 | Identify the report as a systematic review, meta-analysis, or both:  **“a systematic literature review”** | Line 5 |
| **ABSTRACT** | | |  |
| Structured summary | 2 | Provide a structured summary including, as applicable: background; objectives; data sources; study eligibility criteria, participants, and interventions; study appraisal and synthesis methods; results; limitations; conclusions and implications of key findings; systematic review registration number.  **“Neglected zoonotic diseases (NZDs) have a significant impact on the livelihoods of the world’s poorest populations…”**  **“This review looked to identify existing evidence and knowledge gaps regarding animal influence on the effectiveness of WASH interventions for disease control in low and middle income countries.”**  **“Studies from three databases (Medline, Web of Science, Global Health)…”**  **“articles required to show linkages between animal presence and WASH elements at the household level”**  **“results narratively synthesised”**  **“Only two studies out of 7588 met the inclusion criteria” - “a link between animal influence and WASH measures was reinforced, and a lack of articles looking at this interaction identified”**  **“The review could have been improved with more time availability to perform and a second reviewer”**  **“The dearth of studies examining animal-WASH interactions is explained by the difficulties associated with studying environmental interventions…” – “Further specific research with a holistic One Health approach that considers impact of animal husbandry practices is advised…”** | Lines 28 to 49 |
| **INTRODUCTION** | | |  |
| Rationale | 3 | Describe the rationale for the review in the context of what is already known.  **“Neglected tropical diseases (NTDs) are estimated to affect over a billion people around the world, particularly those with least resources, access to health care, good nutrition, clean water and sanitation facilities”**  **“The new global development framework enshrined in the Global Goals of the United Nations’ 2030 Agenda for Sustainable Development sets out a holistic approach to poverty, inequalities, health and the environment…”**  **“Several of the NTDs are zoonotic diseases – infections transmitted between animals and humans, and are therefore referred to as Neglected Zoonotic Diseases (NZDs)”**  **“The need for intersectoral control measures is especially evident in low income countries”**  **“In poor, rural settings, smallholder animal production is dominant, and hence human and animal interaction within the household is more common”**  **“Water, sanitation and hygiene (WASH) programmes can plausibly contribute to control of zoonotic disease given the knowledge about transmission cycles”**  **“No systematic research has been done to date on the impact of demand-side sanitation programmes on NZDs transmission”**  **“As mentioned in the WHO WASH and NTDs Strategy , and as several authors argue , it is necessary to gather more information regarding WASH-related interventions and disease burden reduction”** | Lines 69 to 196 |
| Objectives | 4 | Provide an explicit statement of questions being addressed with reference to participants, interventions, comparisons, outcomes, and study design (PICOS).  **“…to identify the effectiveness of water, hygiene and sanitation measures relative to animal presence in the household for zoonotic disease control in low and middle income countries; to identify gaps of knowledge regarding this topic; and to identify the amount and type of studies looking at this particular interaction.”** | Lines 198 to 201 |
| **METHODS** | | |  |
| Protocol and registration | 5 | Indicate if a review protocol exists, if and where it can be accessed (e.g., Web address), and, if available, provide registration information including registration number.  **“A review protocol was designed…”**  **“The complete protocol can be found in Text S1.”** | Lines 205 to 210 |
| Eligibility criteria | 6 | Specify study characteristics (e.g., PICOS, length of follow-up) and report characteristics (e.g., years considered, language, publication status) used as criteria for eligibility, giving rationale.  **Adapted from S1-File: “Randomised control trials, control trials, quasi-experimental trials, cohort studies, case-control studies, cross-sectional studies, case-report studies”.**  **“Only published studies will be considered for this review. The existent lack of information available regarding animal presence in the household and sanitation program-effectiveness requires the researching team to include all the types of study mentioned above”**  **“The study will focus on human population in rural communities of low and middle income countries in close contact with animals, mainly those cases where animals are present in the household but also those in which animals are kept close to the house and the water, sanitation and human/household waste disposal facilities.”**  **Adapted from S1-File: “Types of outcomes: Primary, including human burden of disease (morbidity, mortality, DALYs, QUALYS) and animal burden of disease (Morbidity, mortality, production losses); Secondary: Risk factors, knowledge gaps, adverse effects, hygiene promotion programme acceptance/efficacy”** | S1-File, page 1 |
| Information sources | 7 | Describe all information sources (e.g., databases with dates of coverage, contact with study authors to identify additional studies) in the search and date last searched.  **“Three databases were used: Medline, Web of Science and Global Health… The three databases were systematically searched for publications dating 1980 to 30^th^ April 2016.”** | Lines 213 to 216 |
| Search | 8 | Present full electronic search strategy for at least one database, including any limits used, such that it could be repeated.  **“The search terms used were chosen based on other WASH literature reviews and expert advice, then divided into four pools:**  **a) Water, hygiene and sanitation: *{[latrine], [toilet], [water], [water supply], [water treatment], [education], [borehole], [standpipe], [rainwater], [sanitary engineering], [pit], [open defecation], [open urination], [shower laundry], [hygiene], [detergent], [soap], [risk factor], [excre*], [faec*], [fecal], [feces], [hand washing], [handwashing], [waste management], [waste disposal]}***  **b) Animals: *{[horse], [pig], [chicken], [turkey], [cow], [dog], [cat], [bovine], [ovine], [porcine], [poultry], [corralling], [farming], [buffalo]}***  **c) Disease: *{[ntds], [nzd], [neglected zoonotic disease], [ntd], [neglected tropical disease], [taenia solium], [cysticercosis], [taeniasis], [pig tapeworm], [trypanosom*], [hat], [nagana], [echinococc*], [hydatidosis], [schistosom*], [snail fever], [foodborne trematod*], [fbt], [chlonorch*], [distomatosis], [liver rot], [opisthorch*], [paragonim*], [lung fluke], [toxoplasm*], [cryptosporid*], [crypto*], [brucell*], [anthrax], [anthracis], [leptospir*], [shigell*], [Escherichia coli], [mycobacterium bovis], [m. bovis]}***  **d) Location: The location terms consisted of the names of all the countries included in the High-Middle, Low-Middle and Low Income countries as defined by the World Bank.**  **The terms amongst pools were combined by the Boolean “OR”, while those between pools were combined by the Boolean “AND”.**  **Diseases chosen for the terms were based on the list of neglected zoonotic diseases described in the WHO NTDs Roadmap . The results obtained were sorted by “author” in descending order.”** | Lines 218 to 242 |
| Study selection | 9 | State the process for selecting studies (i.e., screening, eligibility, included in systematic review, and, if applicable, included in the meta-analysis).  **“For the first stage, title and abstract screening, studies were included if the abstract mentioned the zoonotic disease search terms together with a water, hygiene or sanitation search term, if a full text version was available and if the article was published in English or Spanish.”**  **“In this second stage, articles not quantifying burden of disease in human or animal populations, not analysing the role of animals in zoonosis transmission in relation to WASH measures, or not meeting the quality check described in the protocol, were excluded…”**  **“The selected studies were then checked for quality in the final stage.”**  **“Due to the small number of studies that were selected based on the criteria, quality control was not used for further exclusion.”** | Lines 245 to 247; 251 to 253; 255 to 256; 261 to 262 |
| Data collection process | 10 | Describe method of data extraction from reports (e.g., piloted forms, independently, in duplicate) and any processes for obtaining and confirming data from investigators.  **“Articles included in the full text review were subjected to data extraction based on the protocol…”**  **From S1 file: “Data will be extracted using a predesigned data extraction form. This will be a simple text based form in Word. The data will be entered on to the form to facilitate summarisation and writing of the final report. Sample data extraction form:”** | Line 265 and S1 File (Page 6) |
| Data items | 11 | List and define all variables for which data were sought (e.g., PICOS, funding sources) and any assumptions and simplifications made.  **“…special attention to the study population regarding burden of disease, the diagnostic method used, the WASH measures in place, description of animal presence within the household, and the statistical analysis approach taken by the study.”**  **Adapted from S1 File: “Participants (number, age, ethnicity, animal species, disease, co-morbidities); Intervention/factors (main intervention, secondary interventions, animal factors, human factors); Outcome (Primary outcome: human burden, animal burden; Secondary outcomes: learning/correct usage, knowledge gaps, risk factors)”** | Lines 266 to 268 and S1 File (Page 7) |
| Risk of bias in individual studies | 12 | Describe methods used for assessing risk of bias of individual studies (including specification of whether this was done at the study or outcome level), and how this information is to be used in any data synthesis.  **“Due to the small number of studies that were selected based on the criteria, quality control was not used for further exclusion.”**  **“…a narrative approach was chosen for addressing data synthesis.”** | Lines 261 to 262 and 270 to 271 |
| Summary measures | 13 | State the principal summary measures (e.g., risk ratio, difference in means).  **“…special attention to the study population regarding burden of disease, the diagnostic method used, the WASH measures in place, description of animal presence within the household, and the statistical analysis approach taken by the study.”**  **“…a narrative approach was chosen for addressing data synthesis.”** | Lines 266 to 268 and 270 to 271 |
| Synthesis of results | 14 | Describe the methods of handling data and combining results of studies, if done, including measures of consistency (e.g., I^2^) for each meta-analysis.  **“Due to the consideration of various types of studies in the inclusion criteria and the expected low count of final studies making the last selection, pooling was not deemed possible. Therefore, a narrative approach was chosen for addressing data synthesis.”** | Lines 268 to 270 |

| **Section/topic** | **#** | **Checklist item** | **Reported on page #** |
| --- | --- | --- | --- |
| Risk of bias across studies | 15 | Specify any assessment of risk of bias that may affect the cumulative evidence (e.g., publication bias, selective reporting within studies).  **“…pooling was not deemed possible.”** | Line 270 |
| Additional analyses | 16 | Describe methods of additional analyses (e.g., sensitivity or subgroup analyses, meta-regression), if done, indicating which were pre-specified.  **“Due to the low count of studies included in the final review, the 64 articles studied in this phase were summarised in the form of tables that show the preferences of researchers when addressing WASH and NZDs.”** | Lines 288 to 290 |
| **RESULTS** | | |  |
| Study selection | 17 | Give numbers of studies screened, assessed for eligibility, and included in the review, with reasons for exclusions at each stage, ideally with a flow diagram.  **“Seven thousand five hundred and eighty-eight (n=7588) studies where obtained after introducing the search terms into the three databases (Figure 1).”**  **“Screening of titles and abstracts retrieved a total of 80 studies (n=80) meeting the inclusion criteria for the first stage of the review:”**  **“The total number of articles selected for the next stage of the review was 64.”**  **“After data extraction and analysis, two articles, met the final inclusion criteria as set out in the protocol.”** | Lines 275 to 276; 275 to 281; 287 to 288 |
| Study characteristics | 18 | For each study, present characteristics for which data were extracted (e.g., study size, PICOS, follow-up period) and provide the citations.  **“Table 1: Identified studies results table summary” – Extracted from table “First author, year, location, study design, species, participants, disease, diagnostic, WASH component, animal component, data analysis”.** | Line 309 |
| Risk of bias within studies | 19 | Present data on risk of bias of each study and, if available, any outcome level assessment (see item 12).  **“a multiple correspondence analysis and a hierarchical clustering of several components”**  **“Three clusters were identified, one referential (*cluster 1*)”**  **“Data regarding pig seropositivity was not clustered…”**  **“There was no randomization in village selection or house selection…”**  **“The number of respondets and piga samples increased from pre- to post-intervention.”** | Lines 326 to 328; 334 to 335; 350 to 351; 359 to 360; 372 to 373 |
| Results of individual studies | 20 | For all outcomes considered (benefits or harms), present, for each study: (a) simple summary data for each intervention group (b) effect estimates and confidence intervals, ideally with a forest plot.  **“Average prevalence in humans in each province was: 4.9% and 4.7% for JEV; 48.6% and 77.7% for HEV; 59% and 40.5% for *Trichinella spiralis*; 2.3% and 2.9% for *Taenia spp*.; 6.1% and 1.5% for Cysticercosis.”**  **“…HEV had an OR of prevalence of infection of 2.18 for cluster 2 and 2.30 for cluster 3. *Trichinella spiralis* was less likely to be found in clusters 2 and 3 than 1, with ORs of 0.52 and 0.42 respectively, in contrast with positive reactivity to *Taenia spp.*, with ORs of 2.76 for cluster 2 and 3.38 for cluster 3…”**  **“Table 4: Variables and Odds-Ratio for the diseases studied.”**  **“The prevalence pre-intervention was 13.5% (6.8-20.1, 95% CI), compared to a value of 16.4% (12-20.8, 95% CI)…”**  **Note: results of the two final studies included were summarised narratively, without clustered synthesis.** | Lines 328 to 330; 345 to 348; 353; 361 to 362 |
| Synthesis of results | 21 | Present results of each meta-analysis done, including confidence intervals and measures of consistency.  **Note: results of the two final studies included were summarised narratively, without clustered synthesis.** | Lines 323 to 373 |
| Risk of bias across studies | 22 | Present results of any assessment of risk of bias across studies (see Item 15).  **Note: no pooling was possible in the presentation of results due to the diverse nature of the studies identified.** | Lines 323 to 373 |
| Additional analysis | 23 | Give results of additional analyses, if done (e.g., sensitivity or subgroup analyses, meta-regression [see Item 16]).  **“Due to the low count of studies included in the final review, the 64 articles studied in this phase were summarised in the form of tables that show the preferences of researchers when addressing WASH and NZDs. The complete list with the main data extracted from each one can be found in Table 1…”**  **“Table 1: Identified studies results table summary”**  **“Table 2: Number of papers identified tending to disease and species studied”**  **“Table 3: Number of WASH factors identified and article count”** | Lines 288 to 291; 309; 317; 319 |
| **DISCUSSION** | | |  |
| Summary of evidence | 24 | Summarize the main findings including the strength of evidence for each main outcome; consider their relevance to key groups (e.g., healthcare providers, users, and policy makers).  **“Traditionally, research groups investigating the effectiveness of WASH interventions focus on human factors as positive or negative influences. Similarly, the Veterinary Public Health community focuses more on animal-related factors and disease-transmission routes. The interaction between these two aspects is a research and programming ‘blind spot’”**  **“The study by Holt et al. (2016) compared Odds Ratio of infection in several pig zoonoses between different sanitation and pig contact factors…Pig contact has been described as a risk factor for HEV transmission previously , but according to this study, penning the pigs to avoid household roaming would not make a significant difference…”**  **“The study by Bulaya et al. (2015) showed… Achieving that level of detail in the analysis is an objective for future studies… low latrine usage has been described as a risk factor but also as a recurrent sociocultural problem… poor programme design, lack of follow up or disputes between NGOs and community leaders on logistics, provisions and payments can be a cause for poor latrine construction and maintenance”** | Lines 375 to 511 |
| Limitations | 25 | Discuss limitations at study and outcome level (e.g., risk of bias), and at review-level (e.g., incomplete retrieval of identified research, reporting bias).  **“In the case of *Trichinella*, socioeconomic status acted as a confounder, since the main risk factor is pork consumption…”**  **“…designing impact studies on water, sanitation and hygiene and retrieving significant results is a recurrent challenge for the scientific community: Randomised controlled trials are rarely free from bias, while observational studies usually lack a large enough study population or result significance.”**  **“The study did not specify whether the newly built latrines resulted in safe separation of humans and animals from faeces.”**  **“One of the limitations of the review was the non-inclusion of rodent species in the study.”**  **“To further optimise the systematic review, a second reviewer would have performed the search and selection and compared results.”** | Lines 411 to 412; 434 to 437; 456 to 458; 501 to 511 |
| Conclusions | 26 | Provide a general interpretation of the results in the context of other evidence, and implications for future research.  **“This systematic review demonstrated the relevance of human-animal interaction within the household for sustained disease transmission of NZDs. It also shows the significant lack of specific studies tending to the effect of animals on WASH programmes’ effectiveness for zoonotic disease control.”**  **“Further research should be undertaken regarding the influence of animals in WASH programmes…”** | Lines 513 to 528 |
| **FUNDING** | | |  |
| Funding | 27 | Describe sources of funding for the systematic review and other support (e.g., supply of data); role of funders for the systematic review.  **“This work was supported by the Royal Veterinary College.”** | Line 531 |

*From:*  Moher D, Liberati A, Tetzlaff J, Altman DG, The PRISMA Group (2009). Preferred Reporting Items for Systematic Reviews and Meta-Analyses: The PRISMA Statement. PLoS Med 6(7): e1000097. doi:10.1371/journal.pmed1000097

For more information, visit: **www.prisma-statement.org**.
