# Supplementary material for: Animal influence on water, sanitation and hygiene measures for zoonosis control at the household level: A systematic literature review
Source: PLoS Negl Trop Dis. 2018 Jul 12;12(7):e0006619. doi: 10.1371/journal.pntd.0006619 (PMC6057674; doi:10.1371/journal.pntd.0006619)
Supplement: S2 Checklist — (DOC) [file pntd.0006619.s003.doc]

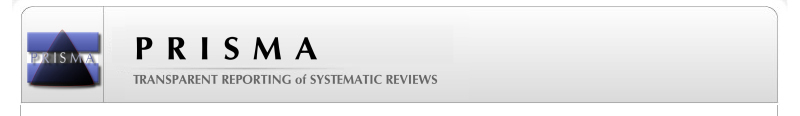
**PRISMA 2009 Flow Diagram**

**Screening**

**Included**

**Eligibility**

**Identification**

Records identified through database searching
Medline, Web of Science, Global Health

(n = 7588)

**Excluded based on title/abstract (n=7508)**
Non-zoonotic disease

No sanitation/hygiene component mentioned

Full text not available

Letter or review

Language not English/Spanish

Records screened
(n = 80)

**Records excluded (n = 16)**

Duplicated publication (n=13)

Full-text impossible to retrieve (n=3)

Full-text articles assessed for eligibility
(n = 64)

**Full-text articles excluded (n = 62)**

No data regarding burden of disease (n=10)

Animal, Sanitation/Hygiene , Human components not isolated or clustered (n=52)

Studies included in qualitative synthesis
(n = 2)
